# Expression of *Agrobacterium* Homolog Genes Encoding T-complex Recruiting Protein under Virulence Induction Conditions

**DOI:** 10.3389/fmicb.2015.01379

**Published:** 2015-12-02

**Authors:** Jing Yang, Meixia Wu, Xin Zhang, Minliang Guo, Zhiwei Huang

**Affiliations:** College of Bioscience and Biotechnology, Yangzhou UniversityYangzhou, China

**Keywords:** *Agrobacterium*, virD2-binding protein (VBP), virulence induction, gene expression, T-complex recruiting protein

## Abstract

The proteins encoded by three Agrobacterial genes, *atu5117, atu4860*, and *atu4856*, are highly homologous to each other in amino acid sequence. All three proteins can bind to VirD2 and are named VBP1, VBP2, and VBP3 (VirD2-binding protein), respectively. VBP is involved in T-DNA transfer by recruiting the T-complex from the cytosol to the polar transport apparatus T4SS (type IV secretion system) and is defined as the “T-complex recruiting protein.” However, it remains unknown how these three homologous genes co-exist in a relatively small prokaryotic genome. To understand whether these three homologous genes are expressed differentially under virulence induction conditions, we examined the effects of virulence induction conditions, including various pH values, temperatures and acetosyringone (AS, an effective virulence inducer to *Agrobacterium tumefaciens*) concentrations, on the expression of the three VBP-encoding genes. Our data showed that *vbp*1 (*atu5117*) and *vbp3* (*atu4856*) maintained constant expression under the tested induction conditions, whereas the expression of *vbp2* (*atu4860*) was affected by the conditions. Culture conditions favorable to the expression of *vbp2* differed from the reported induction conditions for other virulence proteins. In particular, the pH value was a crucial factor for the expression of *vbp2*. In addition, the deletion of *vbp1* affected the expression of *vbp2*. Taken together, these results suggest that the mechanisms regulating the expression of these three homologous genes are different from the virulence induction mechanism and that VBP homologs are presumably involved in other biological processes in addition to T-complex recruitment.

## Introduction

*Agrobacterium tumefaciens* is a well-known phytopathogen that causes crown gall tumor disease in various dicotyledonous plants. Pathogenicity is achieved by the transfer of a T-DNA fragment from the bacterial tumor-inducing (Ti) plasmid into host cells, genetically transforming the host. *Agrobacterium* uses the VirB/D4 T4SS to transfer the T-DNA in the form of a VirD2-T-DNA nucleoprotein complex (called T-complex; Guo et al., 2011; Pacurar et al., 2011; Chandran, 2013; Kado, 2014). The T-DNA transfer process has been largely described, and accumulating data have revealed that many Vir proteins are involved in T-DNA transfer. VirD2, one of the Vir proteins, is a relaxase that can cleave the bottom strand of the T-DNA and covalently attach to the 5′ end of the single-stranded T-DNA, thereby forming the VirD2-T-DNA nucleoprotein complex. In 2007, we used VirD2 as a pull-down bait to identify a VBP; (Guo et al., 2007a). An *Agrobacterial* genome-wide search demonstrated that in addition to the identified VBP-encoding gene *atu5117*, two other *A. tumefaciens* C58 genes, *atu4860* and *atu4856*, can encode two VBP homologs. All three VBP homologs were confirmed to be able to bind VirD2 and thus designated VBP1 (encoded by *atu5117*), VBP2 (encoded by *atu4860*), and VBP3 (encoded by *atu4856*; Guo et al., 2007a,b). Our further investigation showed that VBPs are able to recruit the T-complex from the cytosol to the polar VirB/D4 transport apparatus T4SS (Guo et al., 2007a). Thus, VBPs were also defined as being T-complex recruiting proteins.

Bioinformatics investigations have demonstrated that three VBPs are highly conversed with regard to their functional domains, and all of the VBPs contain a putative nucleotidyltransferase domain near the N-terminus and a putative higher eukaryotes and prokaryotes nucleotide-binding (HEPN) domain near the C-terminus (Guo et al., 2007b; Gao et al., 2013). Structural studies have shown that VBP is a dimer and that the C-terminal HEPN domain is the dimerization domain of VBP. Associated functional studies of the HEPN domain of VBP have demonstrated that the dimerization of VBP is essential for the induction of tumors in plants (Padavannil et al., 2014). Our recent experimental data confirmed that VBP1 (encoded by *atu5117*) is an NTPase that might energize the recruitment of T-complex to the transport site (Gao et al., 2013).

As a ubiquitous soil-born bacterium, *Agrobacterium* has two lifestyles: independent free-living or acting as a phytopathogen. Its pathogenicity is not indispensable for its life cycle (Baek and Shapleigh, 2005; Gao and Lynn, 2005). However, it is currently not understood how a relatively small prokaryotic genome maintains three homologs for a non-essential biological function despite the ability of *Agrobacterium* tumorigenesis to be attenuated only by inactivating all three *vbp* genes. In addition, all three VBPs can complement each other in recruiting the T-complex (Guo et al., 2007a). True genetic redundancy is evolutionarily unstable (Brookfield, 1992; Nowak et al., 1997; Kafri et al., 2008), and bacterial genes that are redundant and not under efficient selection could be rapidly lost (Mira et al., 2001). Intuitively, VBPs may potentially be involved in other biological processes as well as in T-complex recruitment. To explore this possibility, many questions remain unanswered, but one important question is how the expression of three *vbp* genes respond to the virulence induction conditions.

Because VBPs are involved in *Agrobacterium* tumorigenesis, we investigated whether the growth conditions that induce tumorigenesis could affect the expression of the three *vbp* genes. Phenolic compounds and sugar compounds released by wounded plant tissue can be sensed and recognized by *A. tumefaciens*, and induce *Agrobacterium* to express *vir* genes. The induced *Agrobacterium* cells can then transfer the T-DNA to host plant cells, resulting in the formation of a tumor (Pitzschke and Hirt, 2010). Of these compounds, AS is the most effective inducing agent to *Agrobacterium* tumorigenesis. Although the effects of AS concentration, pH and temperature during the inducing process on the transformation efficiency vary in different reports (Baron et al., 2001; Gelvin, 2006; McCullen and Binns, 2006; Sudarshana et al., 2006), it has been proposed that acidic pH (4.8–5.5), moderate temperature (25°C), and a relatively high AS concentration (200 μg/ml) could induce most tumor tissues (Holford et al., 1992; McCullen and Binns, 2006). Thus, we investigated the effects of tumor-inducing conditions, including AS concentration, pH and temperature, on the expression of the three *vbp* genes using western blot analyses. Our data showed that the expression levels of *vbp1* and *vbp3* were nearly unchangeable, independent of the variable induction conditions. However, the expression of *vbp2* was controlled by the culture conditions. Both the temperature and pH optimal for *vbp2* expression were higher than those for virulence induction. The most effective virulence inducer, AS, appears to inhibit the expression of *vbp2*. Among these three tested factors, pH plays an important role in regulating the expression of *vbp2*. The expression of *vbp2* was also affected by *vbp1*-deletion. These results indicate that the expression of three *vbp* genes may be regulated by a novel unknown pathway, which is contradictory from the reported virulence induction pathway. This unknown *vbp*-regulating pathway may be involved in the regulation of *Agrobacterium* tumorigenesis. Taken together, these results also provide additional details for further elucidating the potential versatile functions of VBP homologs.

## Materials and Methods

### Bacterial Strains and Growth Conditions

The strains and plasmids used in the present study are listed in Supplementary Table S1. *Escherichia coli* strains were cultured in Luria broth at 37°C. *A. tumefaciens* strains were cultured in YEP medium, AB-sucrose medium or IB medium at different culture steps, as previously described (please refer to the text; Gelvin, 2006; Guo et al., 2007a). Corresponding antibiotics were added to the culture medium according to Supplementary Table S1.

### DNA Manipulations

The *vbp1* gene fragment was amplified using primers, vbp1-F and vbp1-R (Supplementary Table S1), and cloned into the sites of *Xho* I and *Hind* III endonucleases of the expression vector pRSET-A (The corresponding restriction sites of the *vbp1* gene fragment are underlined in the primers). The resulting plasmid pR-vbp1 was transformed into *E. coli* BL21 (DE3) using the heat shock method (Sambrook et al., 1989). Plasmids expressing *vbp2* and *vbp3* genes were previously constructed (Guo et al., 2007b).

### Heterogeneous Production of Three VBPs

*Escherichia coli* BL21 harboring the corresponding plasmid with the His-tag fused *vbp* gene was grown to an OD_600_ of approximately 0.5 and was subsequently induced by IPTG with a final concentration of 0.3 mM for 1-h to express the *vbp* gene. The induced *E. coli* BL21 cells were harvested by centrifugation (13,000 × *g*, 4°C, 10 min) and washed twice with phosphate-buffered solution (PBS, 10 mM, pH 7.2). Cell pellets from 10 ml of culture were lysed with 2 ml of sodium dodecyl sulfate (SDS)-loading buffer (0.1 M Tris-Cl, pH 6.8, 4% SDS, 0.1% bromophenol blue, 20% glycerol, 200 mM dithiothreitol) and stored at -20°C until further analysis.

### Expression of Three *vbp* Genes in *A. tumefaciens*

To test the responses of *vbp* genes to the virulence induction condition, the preparation of medium for culturing *A. tumefaciens* and the induction culture for different *A. tumefaciens* strains was performed according to ref. 16. For normal virulence induction, cells of different *A. tumefaciens* strains were first inoculated into YEP liquid medium containing appropriate antibiotics and grown overnight at 28°C (while shaking at 250 rpm) and diluted (in the ratio of 1/100, v/v) into AB-sucrose medium containing appropriate antibiotics. After *A. tumefaciens* in AB-sucrose medium grew to OD_600_ approaching 0.8, the cells were harvested by centrifugation (∼3,800 × *g*, 10 min) and washed two times with IB medium. The cell pellets were resuspended in IB medium (pH 5.5) containing 100 μg/ml AS (The cell concentration in IB medium was adjusted to OD_600_ = 0.4) and induced at 25°C for 15–16 h, while shaking at 50 rpm. The induced *Agrobacterium* cells were harvested by centrifugation as described above. *Agrobacterium* cells from 10 ml of IB medium culture were lysed by 1 ml of SDS-loading buffer and stored at -20°C until further protein analysis. Meanwhile, another sample of *Agrobacterium* cells from equal volume of IB medium culture was prepared for the determination of protein concentration in the *Agrobacterium* sample, so that each *Agrobacterium* sample can be adjusted to the same protein concentration for running SDS-polyacrylamide gel electrophoresis (PAGE).

To test the effect of pH on the expression of three *vbp* genes, the pH in IB medium was adjusted to 5.0, 5.5, 6.0, 6.5, 7.0, and 7.5, respectively, and the AS concentration in IB medium was 100 μg/ml. *Agrobacterium* cells were induced in IB medium with different pH and 100 μg/ml AS at 25°C for 15–16 h. To test the effect of temperature on the expression of three *vbp* genes, the pH of the IB medium was adjusted to 5.5; the AS concentration of the IB medium was 100 μg/ml. *Agrobacterium* cells were induced in IB medium at the following five different temperatures: 19, 25, 28, 32, and 37°C. To test the effect of AS concentration on the expression of three *vbp* genes, four different AS concentrations, 0, 50, 100, and 200 μg/ml, were chosen to induce the *Agrobacterium* cells; the pH of the IB medium was adjusted to 5.5. *Agrobacterium* cells were induced in IB medium at different AS concentrations at 25°C for 15–16 h.

### Generation of Antibodies Against Three VBPs

Polyclonal antibodies against three VBPs were supplied by Jinsirui Bio (GenScript-China, Nanjing, China). To obtain the polyclonal antibodies that can discriminate three highly homologous VBPs, peptide fragments from the variable sequences of three VBPs (Supplementary Figure S1) were artificially synthesized and used as antigens to generate polyclonal antibodies against VBPs in rabbits.

### Protein Analysis

Proteins were analyzed using SDS-PAGE and Western blotting analyses. Each protein sample was adjusted to the same concentration. An equal volume of each protein sample was mixed with an equal volume of 2x loading buffer (0.1 M Tris-Cl [pH 6.8], 4% SDS, 0.1% bromophenol blue, 20% glycerol, 200 mM dithiothreitol) and incubated at 100°C for 5 min before loading. After electrophoresis, the gels were stained with Coomassie blue R-250 solution to visualize the protein bands, so that we could further confirm that every sample was loaded equal amount of protein and the protein band pattern of each sample was comparable. For Western blotting, the proteins in gels, which were run in parallel with the Coomassie blue-stained gels, were electrophoretically transferred onto polyvinylidene difluoride membranes (Bio-Rad, Hercules, CA, USA) and detected using the BCIP/NBT alkaline phosphatase color development kit (Beyotime Corp., China) according to the procedure recommended by the manufacturer. Polyclonal antibodies against the variable sequence regions of three VBPs were used as the primary antibodies for detection.

## Results

### Effective Discrimination of Three VBPs

**FIGURE 1:**
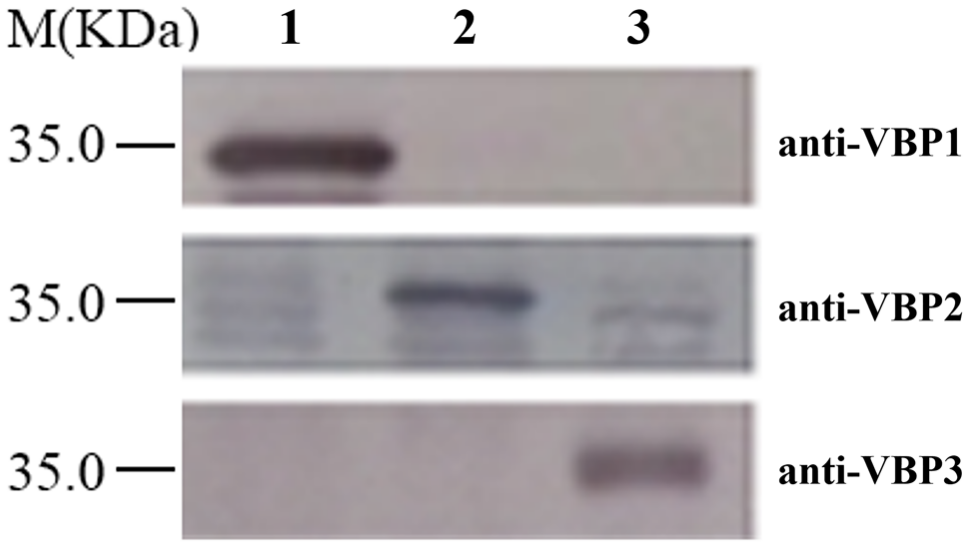
**Discrimination of three virD2-binding proteins (VBPs) by Western blot assay.** Crude extracts from *Escherichia coli* cells expressing different *vbp* genes were separated using SDS-PAGE in the indicated lanes and then analyzed by Western blotting using antibodies against the variable sequence regions of the three VBPs. M: Molecular weight marker. The antibodies used for each assay are listed on the right. Lane 1: crude extracts from *E. coli* cells expressing *vbp1*. Lane 2: crude extracts from *E. coli* cells expressing *vbp2*. Lane 3: crude extracts from *E. coli* cells expressing *vbp3*.

Western blotting is a routine method to assess protein expression. However, the high homology of amino acid sequences of three VBPs (Supplementary Figure S1) suggests that the polyclonal antibody generated by any full-length VBP would most likely cause cross-reaction with each of the three VBPs, resulting in non-specific results. Consequently, the peptide fragments from the variable sequence regions of the three VBPs (283–313 amino acid residues of VBP1, 163–191 amino acid residues of VBP2, and 161–189 amino acid residues of VBP3; Supplementary Figure S1) were artificially synthesized and used as antigens to generate antibodies that could differentiate between the three VBPs. As shown in **Figure 1**, three VBPs heterogeneously produced by *E. coli* could be specifically identified by their corresponding antibodies.

### Expression of Three *vbp* Genes in Wild-type Strain *A. tumefaciens* C58 Differ in Response to *vir* Gene Induction Conditions

*Agrobacterium tumefaciens* C58 is a wild-type strain isolated from a cherry tree tumor; it was completely sequenced in 2001 (Goodner et al., 2001; Wood et al., 2001). The *A. tumefaciens* C58 genome contains a circular chromosome, a linear chromosome, a Ti plasmid pTiC58 and a cryptic megaplasmid pAtC58. In the genome of *A. tumefaciens* C58, *vbp1* is located on the plasmid pAtC58, whereas *vbp2* and *vbp3* are located on the linear chromosome (Guo et al., 2007b). The expression of homologous genes located at different loci may be regulated by different mechanisms. This encourages the exploration of the molecular mechanisms that control the expression of three *vbp* genes.

**FIGURE 2:**
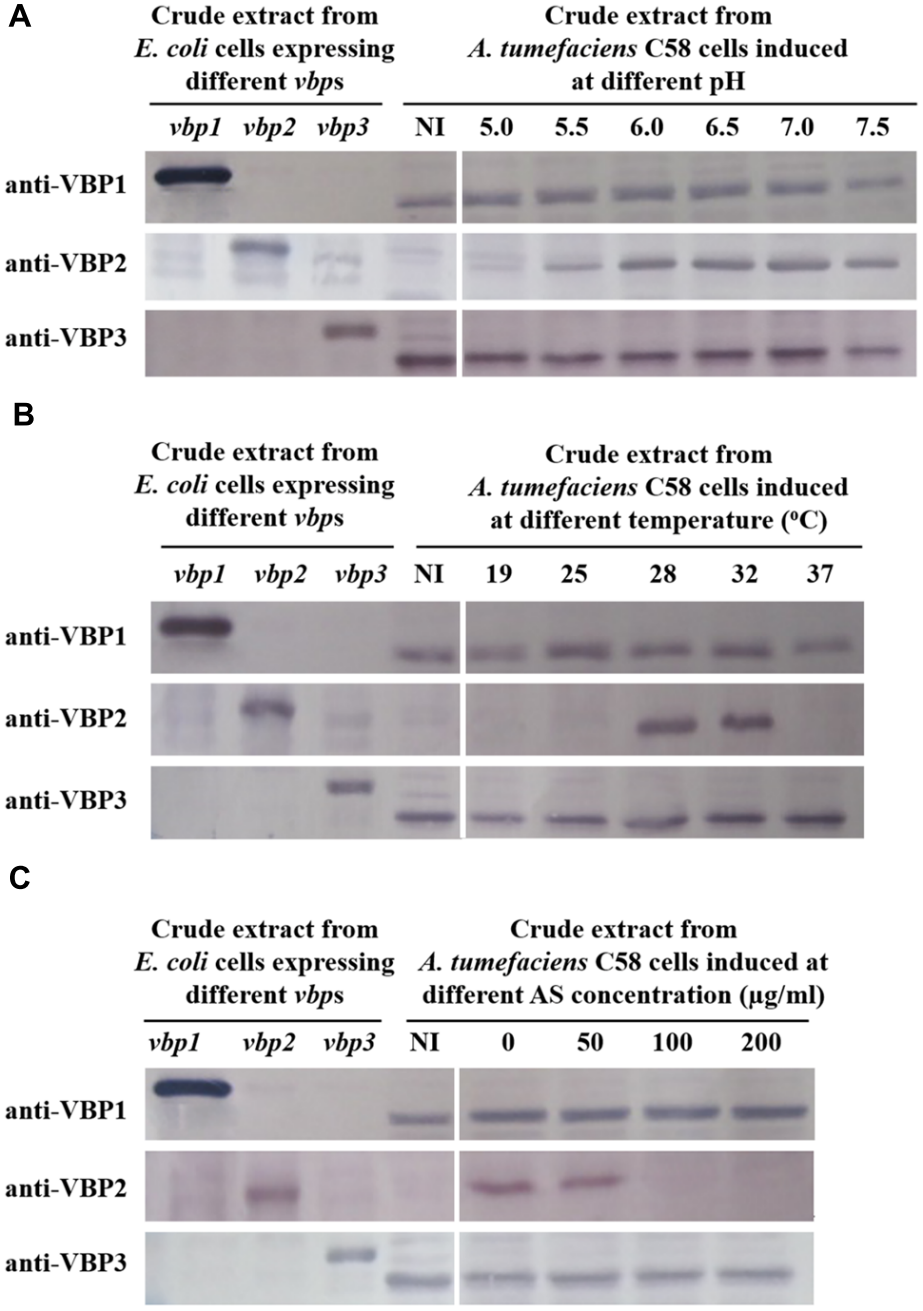
**Expression of three *vbp* genes in *Agrobacterium tumefaciens* strain C58 at different pHs **(A)**, temperatures **(B)** and AS concentrations **(C)**.** *A. tumefaciens* strain C58 cells were induced under the indicated pH, temperature or AS concentration. Crude extracts from the differentially induced C58 cells were separated using SDS-PAGE and then analyzed by Western blotting. Crude extracts from *E. coli* cells expressing different *vbp* genes with His-tag were used as positive controls. The antibodies used in this study are listed on the left. NI: crude extracts from C58 cells not induced in IB medium. **(A)** Effect of pH on the expression of *vbp*s in *A. tumefaciens* C58. The temperature is 25°C and AS concentration is 100 μg/ml. **(B)** Effect of temperature on the expression of *vbp*s in *A. tumefaciens* C58. The pH is 5.5 and AS concentration is 100 μg/ml. **(C)** Effect of AS concentration on the expression of *vbp*s in *A. tumefaciens* C58. The pH is 5.5 and temperature is 25°C. Some differences in protein size of *E. coli* recombinant VBP2 protein (the positive control band in panel C immigrated faster than that in other panels) are likely attributable to prolonged storage.

To test the responses of the three *vbp* genes to *vir* gene induction conditions, *A. tumefaciens* C58 strain was induced by different pHs (5.0, 5.5, 6.0, 6.5, 7.0, and 7.5), AS concentrations (0, 50, 100, and 200 μg/ml) or temperatures (19, 25, 28, 32, and 37°C), and *vbp* gene expression in the differentially induced *Agrobacterium* cells were examined using Western blotting (**Figure 2**). The results showed that the responses to *vir* gene induction conditions differed among the three *vbp* genes. Both *vbp1* and *vbp3* were expressed at any of the tested pH, temperature or AS concentration conditions, whereas the *vbp2* gene was expressed only under some specific induction conditions. Strikingly, *Agrobacterium* cells that were not induced by IB medium did not express *vbp2*, and the optimal pH for *vbp2* expression was within the range of 6.0–7.5, which was significantly higher than that for *vir* gene expression. Furthermore, *vir* gene induction is maximal in the pH range of 5.2–6.0, whereas this pH range was not optimal for the expression of *vbp2* (**Figure 2A**). The results in **Figure 2B** show that *vbp2* was expressed only in the temperature range of 28–32°C, which was higher than the optimal temperature for virulence gene expression. The most effective virulence inducer AS also affected the expression of *vbp2*, but the effect of AS on *vbp2* expression inhibited the expression of *vbp2*. When the concentration of AS was higher than 100 μg/ml, the expression of *vbp2* gene was fully inhibited. Combining all of the results in **Figure 2**, we conclude that the culture conditions optimal for virulence gene induction were not very favorable to the expression of *vbp2*. When comparing the results in **Figure 2**, we found little inconsistency in the expression of *vbp2*. The results in **Figure 2A** show that *vbp2* was weakly expressed under the culture condition of pH 5.5, temperature 25°C and AS concentration 100 μg/ml, but the results shown in both **Figures 2B,C** show that *vbp2* was not expressed under the same culture condition. This slight difference for *vbp2* expression suggested that one of the three factors (pH, temperature, and AS concentration) is critical to the expression of *vbp2* and that any slight fluctuation of the key factor under this culture condition could result in the differential expression of *vbp2*. Data (**Figure 2B**) also showed that the expression of the *vbp2* gene was inhibited at a temperature of 37°C, which was higher than the optimal temperature (28–32°C) for the vegetative growth of *Agrobacterium*. In conclusion, these data indicated that the expression of three *vbp* genes were different from each other under the above-tested induction conditions. Both *vbp1* and *vbp3* genes were expressed constantly, whereas the expression of *vbp2* gene was selective and only occurred near a neutral pH, temperature of 28–32°C and AS concentration lower than 100 μg/ml.

### Deletion of the *vbp1* Gene Affects the Regulation of *vbp2* Expression by *vir* Gene Induction Conditions

As the expression of the three *vbp* genes differed under *vir* gene induction conditions, we investigated whether some regulatory relationships exist among these three homologous genes. To examine the effect of *vbp1* deletion on the expression of *vbp2* and *vbp3, Agrobacterium* strain GMI9017, in which the *vbp1* gene was deleted, was grown under the same induction conditions as the *A. tumefaciens* C58 strain.

**FIGURE 3:**
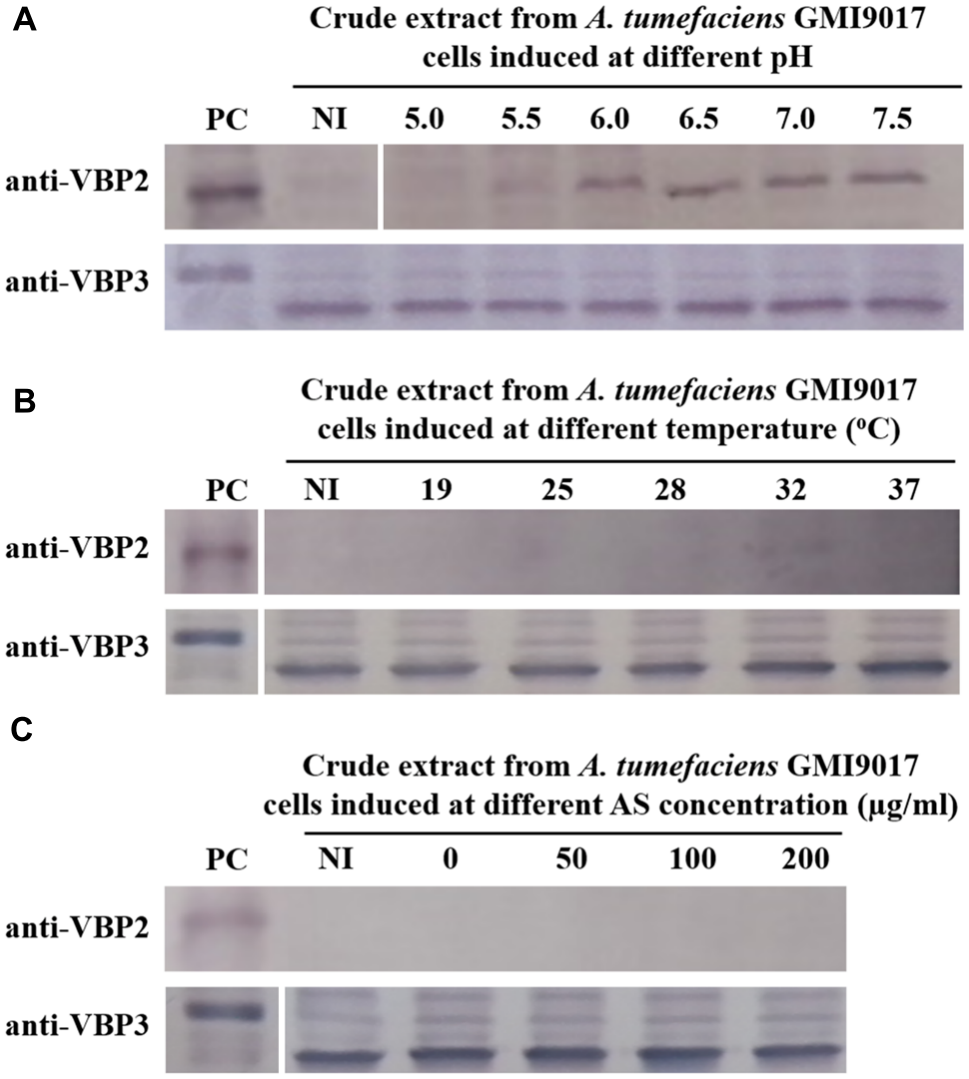
**Expression of *vbp2* and *vbp3* in *A. tumefaciens* strain GMI9017 at different pHs **(A)**, temperatures **(B)**, and AS concentrations **(C)**.** *A. tumefaciens* strain GMI9017 cells were induced under the indicated pH, temperature or AS concentration. Crude extracts from the differentially induced GMI9017 cells were separated using SDS-PAGE and then analyzed by Western blotting. PC, positive control, crude extracts from *E. coli* cells expressing *vbp2* (up) or *vbp3* (down) with His-tag. The antibodies used in this study are listed on the left. NI: crude extracts from GMI9017 cells not induced in IB medium. **(A)** Effect of pH on the expression of *vbp2* and *vbp3* in *A. tumefaciens* GMI9017. The temperature is 25°C and AS concentration is 100 μg/ml. **(B)** Effect of temperature on the expression of *vbp2* and *vbp3* in *A. tumefaciens* GMI9017. The pH is 5.5 and AS concentration is 100 μg/ml. **(C)** Effect of AS concentration on the expression of *vbp2* and *vbp3* in *A. tumefaciens* GMI9017. The pH is 5.5 and temperature is 25°C.

The data showed that deletion of *vbp1* did not affect the expression of *vbp3*. As shown in **Figure 3**, *vbp3* in GMI9017 was expressed under all of the tested induction conditions, similar to C58. However, the expression of *vbp*2 displayed a greater difference in *A. tumefaciens* GMI9017 compared with *A. tumefaciens* C58. The *vbp1*-deleted GMI9017 strain was able to express the *vbp2* gene in the pH range of 6.0–7.5, which was similar to the C58 strain, but it did not express *vbp2* at the acidic pH 5.5, regardless of the temperature and AS concentration (**Figures 3B,C**). Unlike the GMI9017 strain, wild-type strain C58 was able to express *vbp2* at pH 5.5 when grown in a temperature range of 28–32°C or with an AS concentration of less than 50 μg/ml (**Figures 2B,C**). The effects of pH, temperature or AS concentration on the expression of *vbp2* in GMI9017 were different from those in the wild-type strain C58, indicating that deletion of *vbp1* affects the responses of *vbp2* expression to pH, temperature and AS concentration. In other words, *vbp1* is involved in the regulation of *vbp2* expression by pH, temperature and AS concentration, though the mechanism underlying this phenomenon is unclear. When grown at pH 5.5, which is not the pH optimum for *vbp2* expression, GMI9017 did not express *vbp2* at any tested temperature and AS concentration, suggesting that pH may be a crucial external factor in regulating the expression of *vbp2*. Combined with the analysis on the inconsistency of *vbp2* expression at pH 5.5 in C58 (**Figure 2**), we speculated that pH 5.5 might be the pH critical point of *vbp2* expression.

**FIGURE 4:**
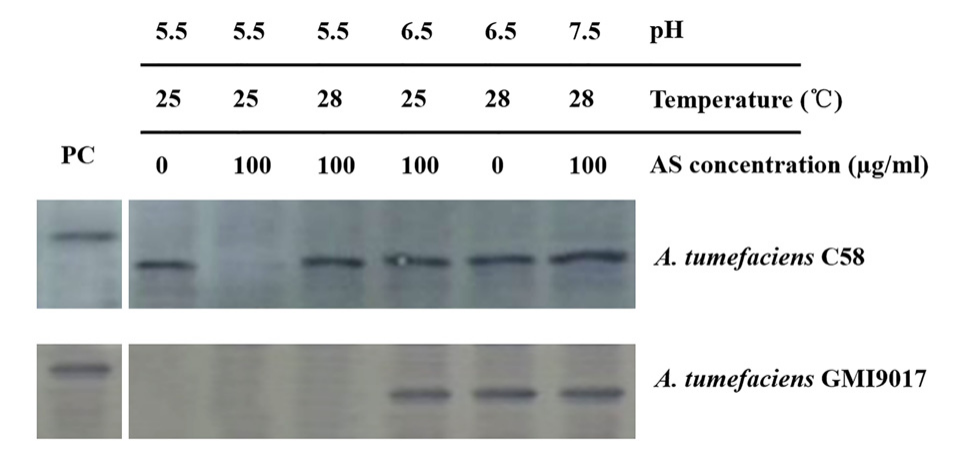
**Comparison of the expression of *vbp2* in *A. tumefaciens* C58 and *A. tumefaciens* GMI9017 at different pHs, temperatures and AS concentrations.** Both the *A. tumefaciens* strain C58 cells and GMI9017 cells were induced in parallel under the indicated pH, temperature or AS concentration. Crude extracts from the differentially induced *Agrobacterium* cells were separated using SDS-PAGE and then analyzed by Western blotting using antibody against VBP2. PC, positive control, crude extract from *E. coli* cells expressing *vbp2* with His-tag.

To eliminate any uncontrollable error that is potentially caused by bacterial culture and induction, the wild-type strain C58 and *vbp1*-deleted strain GMI9017 were induced under different pHs (5.5, 6.5, and 7.5), temperatures (25 and 28°C) or AS concentrations (0 and 100 μg/ml) in parallel. As expected, the results shown in **Figure 4** were consistent with the results in **Figures 2** and **3**. This further confirmed that *vbp1* deletion affected the responses of *vbp2* expression to *vir* gene induction conditions (pH, temperature and AS concentration).

### The Expression of *vbp3* Gene is Not Affected by the Double Deletion of *vbp1* and *vbp2*

**FIGURE 5:**
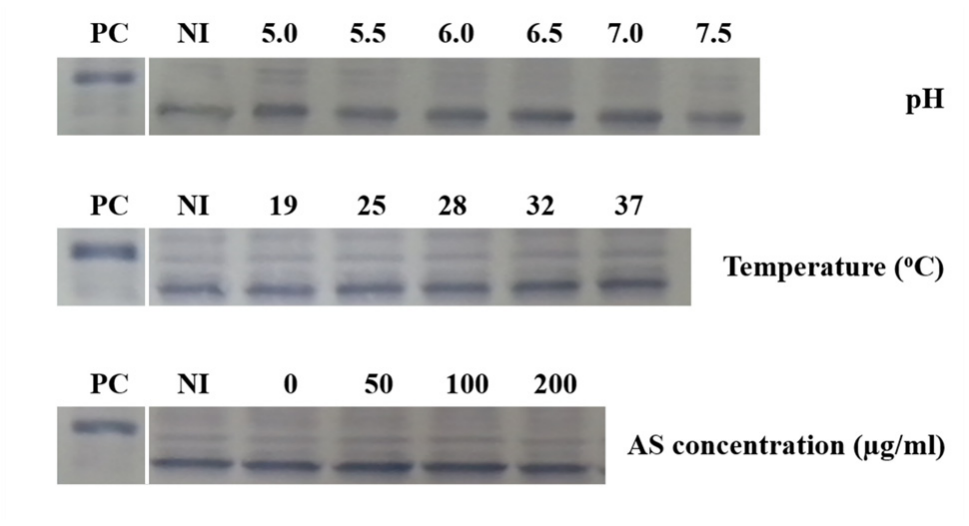
**Expression of *vbp3* in the double-mutant (*vbp1* and *vbp2*) strain GMV12 at different pHs, temperatures and AS concentrations.** *A. tumefaciens* strain GMV12 cells were induced under the indicated pH, temperature or AS concentration. Crude extracts from the differentially induced GMV12 cells were separated using SDS-PAGE and then analyzed by Western blotting with antibody against VBP3. PC, positive control, crude extract from *E. coli* cells expressing *vbp3* with His-tag. NI: crude extracts from GMV12 cells not induced in IB medium.

Although the expression of *vbp3* is not affected by the *vir* gene induction conditions and deletion of *vbp1*, we determined whether a double deletion of *vbp1* and *vbp2* affects the expression of *vbp3*. Thus, we examined the expression of *vbp3* in a double-mutant strain of *vbp1* and *vbp2*, GMV12 under different pHs, temperatures or AS concentrations. No significant change was observed in the expression level of the *vbp3* gene of the GMV12 strain under all of the tested *vir* gene-inducing conditions (**Figure 5**), indicating that in addition to the virulence induction conditions, the homologous genes *vbp1* and *vbp2* do not regulate the expression of *vbp3*.

## Discussion

The responses of three VBP-encoding genes to pH, temperature, and AS concentration are very different from the responses of other reported virulence genes to these three virulence induction factors. According to our data, both *vbp1* and *vbp3* could be expressed ubiquitously, despite varying induction conditions, indicating that neither of these genes are affected by well-known virulence induction conditions. However, *vbp2* showed selective expression under diverse induction conditions. Importantly, pH is a crucial factor for the expression of *vbp2* and is favorable near 7.0. The *vbp2* promoter region was subcloned in front of a promoterless *egfp* gene, and the expression of the reporting gene *egfp* further confirmed the response of the *vbp2* promoter to pH (unpublished data). However, the pH optimal for the expression of virulence genes is approximately 5.5. In *A. tumefaciens*, the reported virulence genes that were induced by acidic conditions (pH 5.5) include all virulence genes located on the Ti plasmid, chromosome virulence genes (*chvG* and *chvI*), and some other genes involved in tumorigenesis, such as *katA* and *aopB* (Xu and Pan, 2000; Jia et al., 2002; Li et al., 2002; Yuan et al., 2008). The induction of *vir* genes by acidic conditions is regulated by a two-component system VirA-VirG. VirA perceives acidic, phenolic or monosaccharide signals, processes all these signals and finally phosphorylates VirG. Subsequently, phosphorylated VirG binds to a conserved 12-bp AT-rich sequence (*vir* box) in the promoter regions of all *vir* genes and initiates their transcription (Pazour and Das, 1990; Yuan et al., 2008). DNA sequence analysis revealed no “*vir* box” in the promoter regions of *vbp* genes, indicating that the *vbp* promoters cannot bind phosphorylated VirG. These findings suggest that *vbp* transcription is not regulated by VirA-VirG-controlling acidic induction. This silica prediction is consistent with our experimental data. In addition, a recent study on the transcriptome of *A. tumefaciens* in response to acidic conditions did not demonstrate that *vbp* transcription was significantly affected by acidic conditions and a deep sequence analysis on the promoters of all four replicons of *A. tumefaciens* did not identify any small RNA targeting sequence in *vbp* gene promoters (Yuan et al., 2008; Wilms et al., 2012). Thus, we conclude that the expression of *vbp1* and *vbp3* is not regulated by *vir*-inducing conditions and that the expression of *vbp2* is regulated by a mechanism different from the reported pH-regulating mechanism. The molecular mechanism regulating the expression of *vbp2* is requires further study.

VBP proteins are able to recruit the VirD2-T-DNA complex to the T4SS apparatus and are thus important for the pathogenicity of *A. tumefaciens* (Guo et al., 2007a). The similarity of three VBPs in the amino acid sequence is over 70% and three VBPs are redundant for the function of T-complex recruitment. However, from an evolutionary perspective, functional overlaps of homologous proteins are inherently unstable. If a protein’s function can be fully compensated for by a redundant homolog, then the mutations in the protein-encoding gene would have no effect on the phenotype. Consequently, such mutations would not remain, and the redundancy would be gradually eliminated (Lynch and Conery, 2000). Homologous genes can be obtained from duplications and lateral acquisitions. A recent study declared that the secondary chromosome (linear chromosome) of *A. tumefaciens* C58 originated from an intragenomic transfer from the primary chromosome (circular chromosome) to an ancestral plasmid (Slater et al., 2009). Both *vbp2* and *vbp3* genes are located on the linear chromosome, indicating that these two homologous genes were likely to originate from duplication, whereas, the *vbp1* gene is located on the cryptic megaplasmid pAtC58, indicating that *vbp1* was potentially obtained from lateral acquisition. Several homologs inhabit the same genome stably, indicating that either their functions or their *cis*-regulatory motifs have diversified (Gu et al., 2004). The difference between the expression of *vbp2* and *vbp3* genes demonstrated that the *cis*-regulatory motifs of these two homologs have diversified. However, the expression of *vbp1* is very similar to the expression of *vbp3*, implying that both promoter regions of *vbp1* and *vbp3* may have similar *cis*-regulatory motifs. We used several databases^1^ to analyze the promoter regions of *vbp1* and *vbp3*. The analysis showed that both promoter regions of *vbp1* and *vbp3* have the same transcription factor-binding motifs (data not show). The co-inhabitancy of *vbp1* with its homologs in the same genome indicates that the function of *vbp1* has diversified from its homologs. Therefore, these three *vbp* homologs may provide a good example for the research of homologous gene evolution. Recent bioinformatics studies have shown that redundant protein partners are significantly more frequently associated with the essential core proteins of protein-interaction networks (Kafri et al., 2008; Plata and Vitkup, 2014). T-DNA transfer is not necessary for the life cycle of *A. tumefaciens*. Nevertheless, the *A. tumefaciens* mutant of the *vbp* triple-deletion was difficult to construct (Guo et al., 2007b), demonstrating that VBP may be involved in some essential biological process as well as T-complex recruitment. Evidence that the expression of three *vbp* genes is not involved in the expression of other virulence genes suggests that the major function of VBP homologs is not T-complex recruitment. Taken together, these data suggest that redundant VBPs are versatile proteins and are involved in biological processes other than T-complex recruitment. It is possible that VBP1 has diversified to specify T-complex recruitment. However, the biological processes involving VBP require further investigation.

## Author Contributions

MG planned the experiments. JY, MW, XZ, and ZH prepared and performed the experiments. JY, MG, and MW analyzed the data. JY and MG wrote the paper.

## Conflict of Interest Statement

The authors declare that the research was conducted in the absence of any commercial or financial relationships that could be construed as a potential conflict of interest.

## Footnotes

1 http://linux1.softberry.com/; http://www.cbil.upenn.edu; http://pepper.molgenrug.nl

## Abbreviations

AS: acetosyringone
HEPN domain: higher eukaryotes and prokaryotes nucleotide-binding domain
IB medium: inducing broth medium
T4SS: type IV secretion system
Ti plasmid: tumor-inducing plasmid
VBP: VirD2-binding protein
Vir proteins: virulence proteins
