# Supplementary material for: Expression of Agrobacterium Homolog Genes Encoding T-complex Recruiting Protein under Virulence Induction Conditions
Source: Front Microbiol. 2015 Dec 2;6:1379. doi: 10.3389/fmicb.2015.01379 (PMC4667095; doi:10.3389/fmicb.2015.01379)
Supplement: Supplementary file 1 [file Data_Sheet_1.DOCX]

***Supplementary Material***

**Expression of *Agrobacterium* homolog genes encoding T-complex recruiting protein under virulence induction conditions**

**Jing Yang, Meixia Wu, Xin Zhang, Minliang Guo* and Zhiwei Huang**

College of Bioscience and Biotechnology, Yangzhou University, P. R. China

*** Correspondence:** M. Guo, College of Bioscience and Biotechnology, Yangzhou University, Yangzhou City, Jiangsu 225009, P. R. China. E-mail: guoml@yzu.edu.cn

1. **Supplementary Figures and Tables**


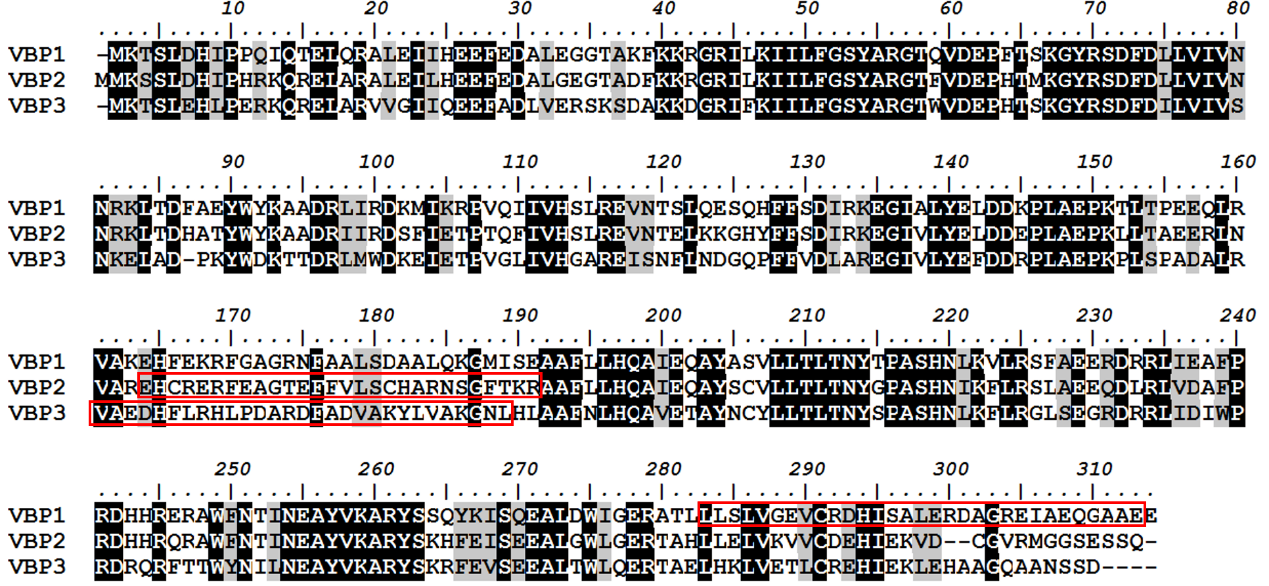


**Supplementary Figure 1. Amino acid sequence alignment of three VBPs.** The numbers on the top show amino residue locations. Identical residues are shaded in black. Similar residues are shaded in grey. The red frames show the specific peptides synthesized for discriminating three VBPs: the 283-313 amino acid residues of VBP1, 163-191 amino acid residues of VBP2, and 161-189 amino acid residues of VBP3. These three peptide fragments were used to generate antibodies against three VBPs.

**Supplementary Table 1. Plasmids and strains used in this study.**

| Primers, plasmids or strains | Description | Source or reference |
| --- | --- | --- |
| **Primers**  vbp1-F  vbp1-R  **Plasmids** | 5'-CCCTCGAGGAAAACATCGCTCGATCATATT-3'  5'-AAAAAGCTTTAGCCCGCTATTCTTCAG-3' | *vbp1* upstream  *vbp1* downstream |
| pRSET-A | pUC-derived expression vector; Amp^r^ | Invitrogen |
| pR-vbp1 | pRSET-A inserted with his-tag fused *vbp1*; Amp^r^ | Present study |
| pR-vbp2 | pRSET-A inserted with his-tag fused *vbp2*; Amp^r^ | Laboratory collection |
| pR-vbp3 | pRSET-A inserted with his-tag fused *vbp3*; Amp^r^ | Laboratory collection |
| **Strains** |  |  |
| *E. coli* DH5α | *endA1 hsdR17* (r^-^ m^+^) *supE44 thi-1 recA1 gyrA* (NalR) *recA1* Δ (*lacZYA-argF*)*U169 deoR* [Ø80Δd*lacZ* ΔM15], host strain for DNA cloning | Laboratory collection |
| *E. coli* BL21(DE3) | F^-^, *omp*T *hsdS_B_* (*r_B_^-^*, *m_B_^-^*), *gal*, *dcm* (DE3), host strain for protein expression | Invitrogen |
| *E. coli* BL21-VBP1 | *E. coli* BL21 expressing his-tag fused *vbp1*; Amp^r^ | Present study |
| *E. coli* BL21-VBP2 | *E. coli* BL21 expressing his-tag fused *vbp2*; Amp^r^ | Laboratory collection |
| *E. coli* BL21-VBP3 | *E. coli* BL21 expressing his-tag fused *vbp3*; Amp^r^ | Laboratory collection |
| *A. tumefaciens* C58 | Wild-type, nopaline-type pTiC58 plasmid | Laboratory collection |
| *A. tumefaciens* GMI9017 | C58 cured of pAtC58; lacking *vbp1*; Sm^r^, Sp^r^, Rf^r^ | (Rosenberg and Huguet, 1984) |
| *A. tumefaciens* GMV12 | Derivative of GMI9017 in which *vbp2* was deleted | Laboratory collection |
| *A. tumefaciens* GMV123 | Derivative of GMI9017 in which *vbp2* and *vbp3* were mutated; Cb^r^ | Laboratory collection |

1. **References^[[1]](#footnote-1)^**

Rosenberg, C., and Huguet, T. (1984) The pAtC58 plasmid of *Agrobacterium tumefaciens* is not essential for tumour induction. *Mol. Gen. Genet.* **196**, 533-536. doi: 10.1007/BF00436205.

1. Provide the doi when available, and ALL complete author names. [↑](#footnote-ref-1)
